# Pyrosequencing 16S rRNA genes of bacteria associated with wild tiger mosquito *Aedes albopictus*: a pilot study

**DOI:** 10.3389/fcimb.2014.00059

**Published:** 2014-05-14

**Authors:** Guillaume Minard, Florence-Hélène Tran, Audrey Dubost, Van Tran-Van, Patrick Mavingui, Claire Valiente Moro

**Affiliations:** Ecologie Microbienne, UMR CNRS 5557, USC INRA 1364, VetAgro Sup, FR41 BioEnvironment and Health, Université de Lyon 1Villeurbanne, France

**Keywords:** high-throughput sequencing, 16S rDNA, bacterial diversity, *Wolbachia*, field-caught mosquitoes

## Abstract

The Asian tiger mosquito *Aedes* (Stegomya) *albopictus* is an invasive species that has spread across the world in the last two decades, showing a great capacity to adapt to contrasting climates and environments. While demonstrated in many insects, the contribution of bacterial symbionts in *Aedes* ecology is a challenging aspect that needs to be investigated. Also some bacterial species have already been identified in *Ae. albopictus* using classical methods, but a more accurate survey of mosquito-associated bacterial diversity is needed to decipher the potential biological functions of bacterial symbionts in mediating or constraining insect adaptation. We surveyed the bacteria associated with field populations of *Ae. albopictus* from Madagascar by pyrosequencing 16S rRNA gene amplicons. Different aspects of amplicon preparation and sequencing depth were tested to optimize the breadth of bacterial diversity identified. The results revealed that all mosquitoes collected from different sites have a bacterial microbiota dominated by a single taxon, *Wolbachia pipientis*, which accounted for about 99% of all 92,615 sequences obtained. As *Ae. albopictus* is known to harbor two *Wolbachia* strains (*w*AlbA and *w*AlbB), a quantitative PCR was used to estimate the relative densities, (i.e., the bacteria-to-host gene ratios) of each strains in individual mosquitoes. Relative densities were between 6.25 × 10^0.01^ and 5.47 × 10^0.1^ for *w*AlbA and between 2.03 × 10^0.1^ and 1.4 × 10^1^ for *w*AlbB. Apart from *Wolbachia*, a total of 31 bacterial taxa were identified at the genus level using different method variations. Diversity index values were low and probably underestimated the true diversity due to the high abundance of *Wolbachia* sequences vastly outnumbering sequences from other taxa. Further studies should implement alternative strategies to specifically discard from analysis any sequences from *Wolbachia*, the dominant endosymbiotic bacterium in *Ae. albopictus* from this area.

## Introduction

Invasive mosquitoes give cause for worldwide concern not only because of their potential ecological impact, but also they are vectors of a wide range of pathogens affecting both humans and animals (Medlock et al., 2012). In particular, the tiger mosquito *Aedes albopictus* has rapidly spread across the world from its native South-East Asia and is considered as one of the world's 100 most dangerous invasive species (Benedict et al., 2007). *Ae. albopictus* was found to be competent for more than 20 arboviruses (Gratz, 2004). *Ae. albopictus* was involved in the transmission of the Chikungunya and Dengue viruses worldwide, including to Europe (Bonilauri et al., 2008; Grandadam et al., 2011; Caron et al., 2012), and was also implicated as a vector of the parasitic nematode *Dirofilaria* (Cancrini et al., 2007).

In light of the recent theory of the holobiont unit, it is now necessary to enlarge our view of how commensal host-bacterial relationships function in insects (Feldhaar, 2011; Minard et al., 2013a). Insects establish numerous types of symbiotic associations ranging from parasitism to mutualism with their microbial communities (Toft and Andersson, 2010). Recent studies have highlighted how bacterial endosymbioses influence evolutionary and ecological processes through effects on insect host biology (Douglas, 2011). However, most examples demonstrate how symbiotic microbiota are essential to phytophagous insect functions such as host plant specialization (Tsuchida et al., 2004), reproduction (Simon et al., 2011), protection against natural enemies (Oliver et al., 2003), and tolerance to environmental stress (Brumin et al., 2011; Feldhaar, 2011). It is therefore necessary to assess how the bacterial microbiota contributes to the biology of hematophagous insects, particularly invasive mosquitoes.

Until now the composition of bacterial communities associated with *Ae. albopictus* has mainly been investigated using classical microbiological and molecular techniques. Various culture-dependent isolation methods were used to identify cultivable bacteria (Zouache et al., 2009; Chouaia et al., 2010; Minard et al., 2013a,b; Valiente Moro et al., 2013). In parallel, non-culture-based methods such as DGGE fingerprinting (Chouaia et al., 2010; Zouache et al., 2011) or taxonomic microarray hybridization (Zouache et al., 2012) were employed. Together these approaches have unraveled some of the bacterial diversity present in both laboratory-reared and field-caught *Ae. albopictus* mosquitoes. However, these relatively low-throughput techniques cannot provide comprehensive coverage of the microbial composition and diversity in mosquito hosts. The recent rapid development of high-throughput sequencing methods has made it possible to detect a deeper level of microbial diversity in animal hosts, it has been demonstrated for symbiotic microbiotas of insect guts (Shi et al., 2010; Fukatsu, 2012). To date very few studies have used Next-Generation Sequencing technologies to explore bacterial microbiota in mosquitoes. Osei-Poku et al. (2012) successfully used pyrosequencing of 16S rRNA genes to investigate the diversity of gut bacterial communities in field-caught mosquitoes and showed that bacterial composition varies greatly between populations. When pyrosequencing was used to determine the composition of the midgut microbiota of the malaria mosquito vector *Anopheles gambiae*, much richer information was obtained than from low-throughout methods (Wang et al., 2011; Boissière et al., 2012).

The goal of the present study was to survey the bacterial diversity associated with *Ae. albopictus* mosquitoes by pyrosequencing 16S rDNA genes. The effect of varying different methodological parameters like those for amplicon preparation and sequencing depth on the bacterial diversity results was assessed.

## Materials and methods

### Mosquito collection

The sampling area and mosquito capture procedure were approved by Madagascar National Parks. Mosquito specimens were collected from various natural breeding sites on the east coast of Madagascar (Toamasina) in the Antsinanana region (18° 8′ 59.64″ S) in December 2010. Butterfly nets were used to catch adult mosquitoes flying near the grass or around the capturers. *Aedes albopictus* specimens were identified using morphological characteristics and pictorial keys (Rueda, 2004) and then female and male mosquitoes were separated. Only non-blooded female mosquitoes were used for the analysis, as previous studies have demonstrated that blood meals have a dynamic effect on bacterial diversity (Wang et al., 2011; Boissière et al., 2012). Mosquitoes were stored in 70% ethanol at −80°C until use.

### Genomic DNA extraction

All DNA extractions were performed in a sterile environment under a laminar hood to avoid contamination. Mosquitoes were surface-disinfected with 70% ethanol and rinsed with sterile water, as previously described (Minard et al., 2013b). Each mosquito was individually crushed using a Bioblock Scientific MM 2000 mill (Retsch, France) in an Eppendorf tube with 5-mm diameter inox beads and 200 μl of extraction buffer (2% hexadecyltrimethyl ammonium bromide, 1.4 M NaCl, 0.02 M EDTA, 0.1 M Tris pH 8.0, 0.2% 2-β-mercaptoethanol) and heated at 60°C for 2 h. Lipids and proteins were extracted with phenol-chloroform-isoamyl alcohol (25:24:1; v/v/v) and then chloroform:isoamyl alcohol (24:1; v/v). DNA was precipitated with isopropyl alcohol and centrifugation at 16,100 g for 30 min at 4°C. DNA pellets were washed with cold ethanol, air-dried, and resuspended in 20 μl TE buffer (2 mM Tris, 1 mM EDTA).

### Primers and PCR amplification

Hypervariable *rrs* gene V5-V6 regions of about 280 bp were amplified with 784F 5′-AGGATTAGATACCCTGGTA-3′ and 1061R 5′-CRRCACGAGCTGACGAC-3′ primers (Andersson et al., 2008). These primers were selected *in silico* with the RDP Probe Match tool according to the following criteria (Cole et al., 2009): hybridization with 94% of sequences belonging to the RDP Bacteria domain database with good quality and ≥1200 bp long with 2 mismatches allowed in primer sequences. In addition, Basic Local Alignment Search Tool (BLAST) was used to check that the chosen primers did not match *Ae. albopictus* 18S and mitochondrial 16S rDNA gene sequences. PCR amplification was performed using 1.75 U of Expand High Fidelity Enzyme Mix (Roche, Switzerland) with 1× Expand High Fidelity Buffer (Roche, Switzerland), 0.06 mg mL^−1^ of T4 gene 32 protein (Roche, France), 0.06 mg mL^−1^ of bovine serum albumin (New England Biolabs, France), 40 μM of dNTP mix, 200 nM of each primer (Invitrogen, France) and 30 ng of template DNA. PCR products were purified with QiaAmp purification kit (Qiagen, France) following the manufacturer's recommendations.

### Preparation of samples for 454 pyrosequencing and bioinformatic processing

**Figure 1:**
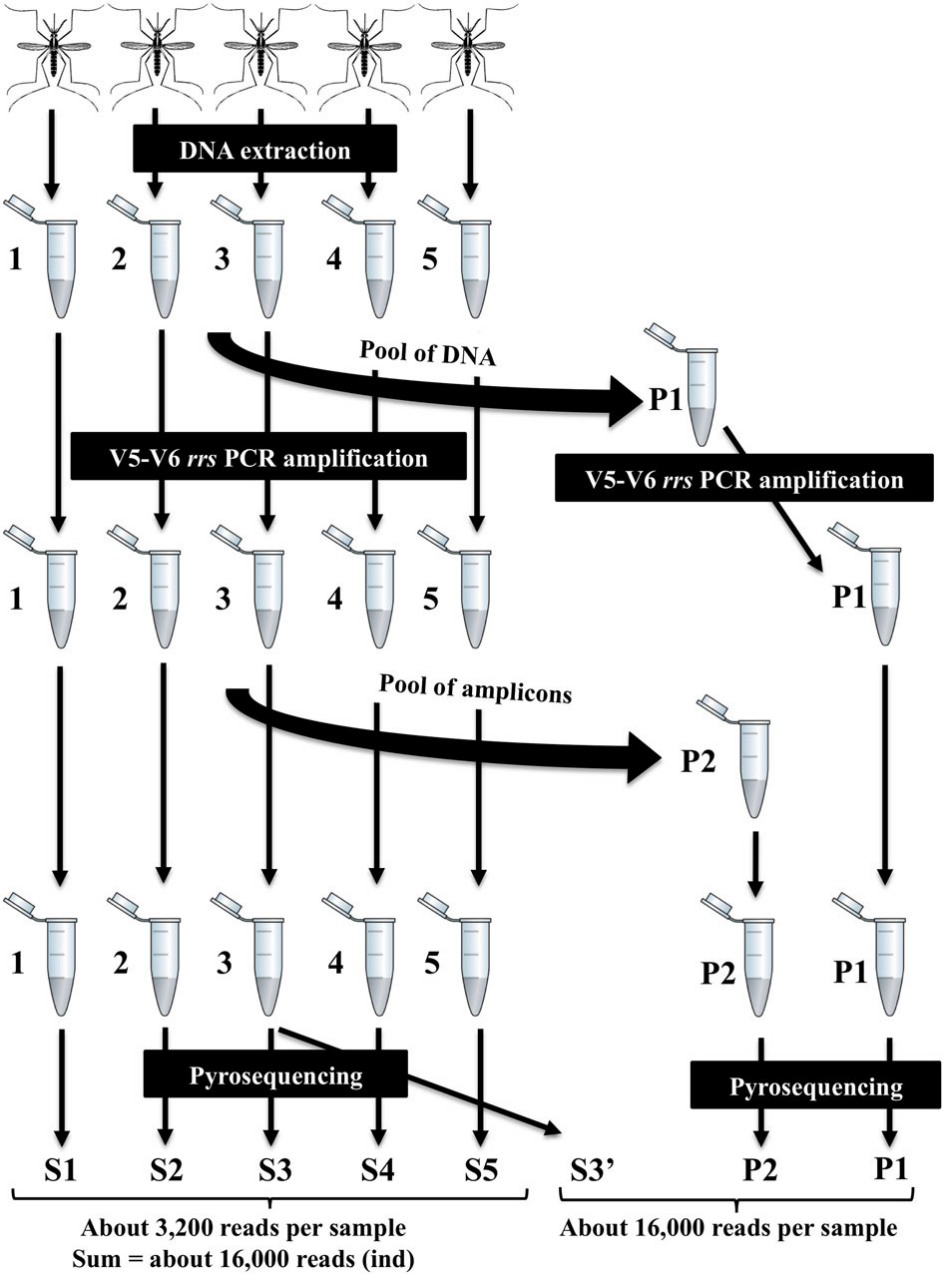
**Experimental approach to test various methods for in-depth exploration of the bacterial diversity associated with *Ae. albopictus* mosquitoes**. Different steps include (1) DNA extraction from individuals, (2) V5-V6 *rrs* PCR amplification by constituting a pool of DNA before amplification (or not), (3) pyrosequencing by pooling PCR amplicons from several individuals (or not). A total of eight V5-V6 *rrs* amplicon libraries were sequenced at two different ranges of about 3200 reads per sample (S1, S2, S3, S4, S5) and 16,000 reads per sample (S3′, P1, P2).

Amplicon tagging and pyrosequencing were performed by a commercial laboratory (DNAvision, Belgium) using the Roche 454 FLX Titanium platform (Roche, Switzerland). Various modalities were tested for in-depth exploration of bacterial diversity (Figure 1) including: (i) constituting a pool of DNA then performing PCR amplification vs. pooling PCR of several amplicons generated from different individual samples; (ii) sequencing a single amplicon vs. a pool of amplicons; and (iii) evaluating the depth of sequencing (number of reads) necessary for the most complete examination of the bacterial diversity. A total of eight *rrs* V5-V6 amplicon libraries were sequenced at two different ranges of about 3200 reads per sample (S1, S2, S3, S4, S5) and 16,000 reads per sample (S3′, P1, P2) (Figure 1). Data were analyzed by first trimming sequence quality with different cutoffs using standard filtering tools in the Mothur package (Schloss et al., 2009). Two errors on primer sequence and one error on barcode sequence were allowed. Sequences shorter than 250 bp were discarded and chimeric sequences were removed using UCHIME and manual analysis (Edgar et al., 2011). Sequences were grouped into operational taxonomic units (OTUs) by clustering at 97% similarity. Singleton reads assigned to OTUs were discarded from the analysis if they were not found in at least two different samples as singletons are likely to be sequencing errors which can lead to an overestimation of diversity. Rarefaction curves were built to estimate sample coverage. Richness, α diversity and β diversity were calculated to compare samples with respectively richness estimators (Chao, Jackknife, Abundance Coverage Estimator), diversity indices (Simpson, Shannon) and Bray–Curtis dissimilarity. Bray–Curtis dissimilarity values were used to construct a neighbor joining tree with the BIONJ algorithm (Gascuel, 1997) using R software (R Development Core Team, 2009). OTU consensus sequences were screened against the SILVA database (release 111) with a 5% dissimilarity cut-off and an *e*-value of 10^−12^. Taxonomic assignment of sequence clusters was performed with local BLASTALL searches. A new sample, named ind, was added to the analysis. This sample was generated by pooling all the reads from each individual sample (S1 to S5) (Figure 1). All sff files were deposited at EMBL European Nucleotide Archive (www.ebi.ac.uk/ena/) under project accession number PRJEB4976.

### Quantification of *Wolbachia*

The relative density of *Wolbachia pipientis* was measured by real-time quantitative PCR specifically targeting the *wsp* genes for each of the *Wolbachia* strains *w*AlbA and *w*AlbB (Zouache et al., 2011). Amplification using the LightCycler LC480 apparatus (Roche) was performed using 5 ng of DNA from a single female mosquito (10 females in total), as previously described (Zouache et al., 2011). Standard curves were constructed using a dilution series (10^1^–10^8^ molecules) of the pQuantAlb plasmid containing *wsp* and *actin* fragments (Tortosa et al., 2008). For each gene, three technical replicates were processed per DNA sample.

## Results

### Bacterial species richness and diversity in mosquitoes

**Figure 2:**
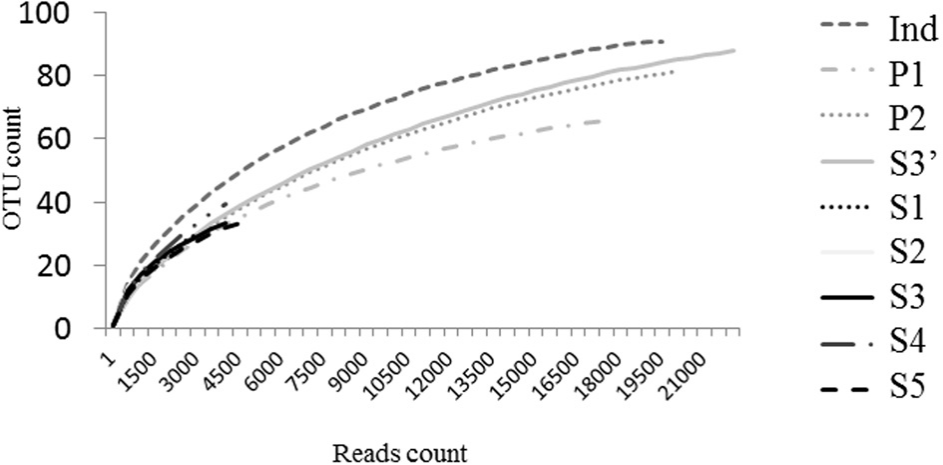
**Rarefaction analysis of bacterial microbiota discovery**. Operational taxonomic units (OTUs) were grouped with a 97% similarity. Rarefaction curves represent the number of new OTUs discovered by sampling without being recaught.

**Figure 3:**
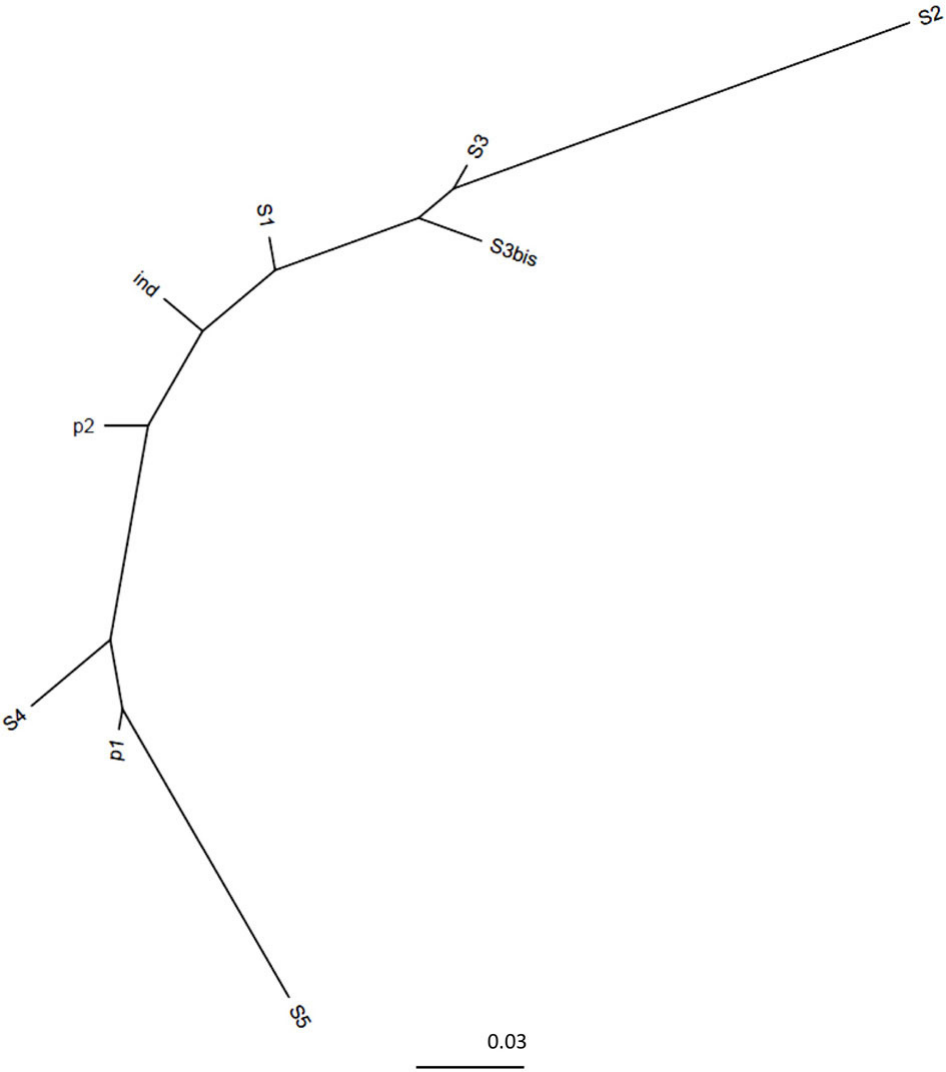
**Dissimilarity distances tree**. The neighbor joining tree represents dissimilarity percentages between samples. It was computed with the Bray–Curtis dissimilarity index using R software.

The V5-V6 regions of bacterial *rrs* genes amplified from female *Ae. albopictus* adults were sequenced by high-throughput pyrosequencing. From the total of 92,615 reads obtained, approximately 79,895 were judged to be of good quality. For samples from individual mosquitoes (S1, S2, S3, S4, S5), read numbers varied from 1583 (S2) to 5048 (S5). For P1, P2, Ind, and S3′, read numbers were 17,584, 20,447, 19,132, and 22,199, respectively. Sequences clustered at 97% similarity into 417 OTUs. The coverage of the sequencing was determined by using rarefaction analysis. The rarefaction curves of samples from single mosquitoes did not reach saturation (Figure 2). On the contrary, saturation was almost attained for the ind pool of individual sample sequences (S1–S5), P1 sequences obtained from amplicons generated from a pool of DNA and P2 sequences obtained from a pool of amplicons (i.e., after amplification of a single sample). The comparison of rarefaction curves between S3 (4276 reads) and S3′ (22,296 reads) clearly indicated that for a single mosquito individual a minimal sequence number close to 16,000 reads is sufficient for a good estimation of sample richness. Indeed, diversity indices Chao1, ACE1, Jackknife for S3 are about half of the respective S3′ values (Table 1). However, the Simpson and Shannon diversity indices of S3 and S3′ were similar. In addition, analyses of Bray–Curtis distances showed a low level of dissimilarity (*BC* = 0.03939) between S3 and S3′. Other dissimilarity scores revealed that overall the P2 and ind pools (*BC* = 0.059085) are more similar to each other than to P1 (Figure 3).

**Table 1:** **Richness and diversity index**.

	**Richness**	**Chao1**	**ACE1**	**Jackknife**	**λ**	**H′**
S1	32	44.0000	94.3809	48.0000	0.5537	0.8149
S2	23	36.7500	48.4876	34.8822	0.4548	0.9647
S3	34	43.7500	60.0596	47.0000	0.5092	0.8708
S4	44	69.3000	133.3393	68.9360	0.6461	0.7645
S5	35	42.8000	48.1620	48.0000	0.8159	0.4642
S3′	88	99.3226	112.2252	115.0000	0.5159	0.8063
P1	66	75.5000	85.8432	86.0000	0.6802	0.6256
P2	82	94.0370	107.1057	108.0000	0.6039	0.7106
ind	91	99.5556	106.7494	113.0000	0.5967	0.8214

*Indexes were calculated for each sample with 3% distant OTUs*.

### *Wolbachia* sequences are the predominant 16S rDNA amplicons

**Figure 4:**
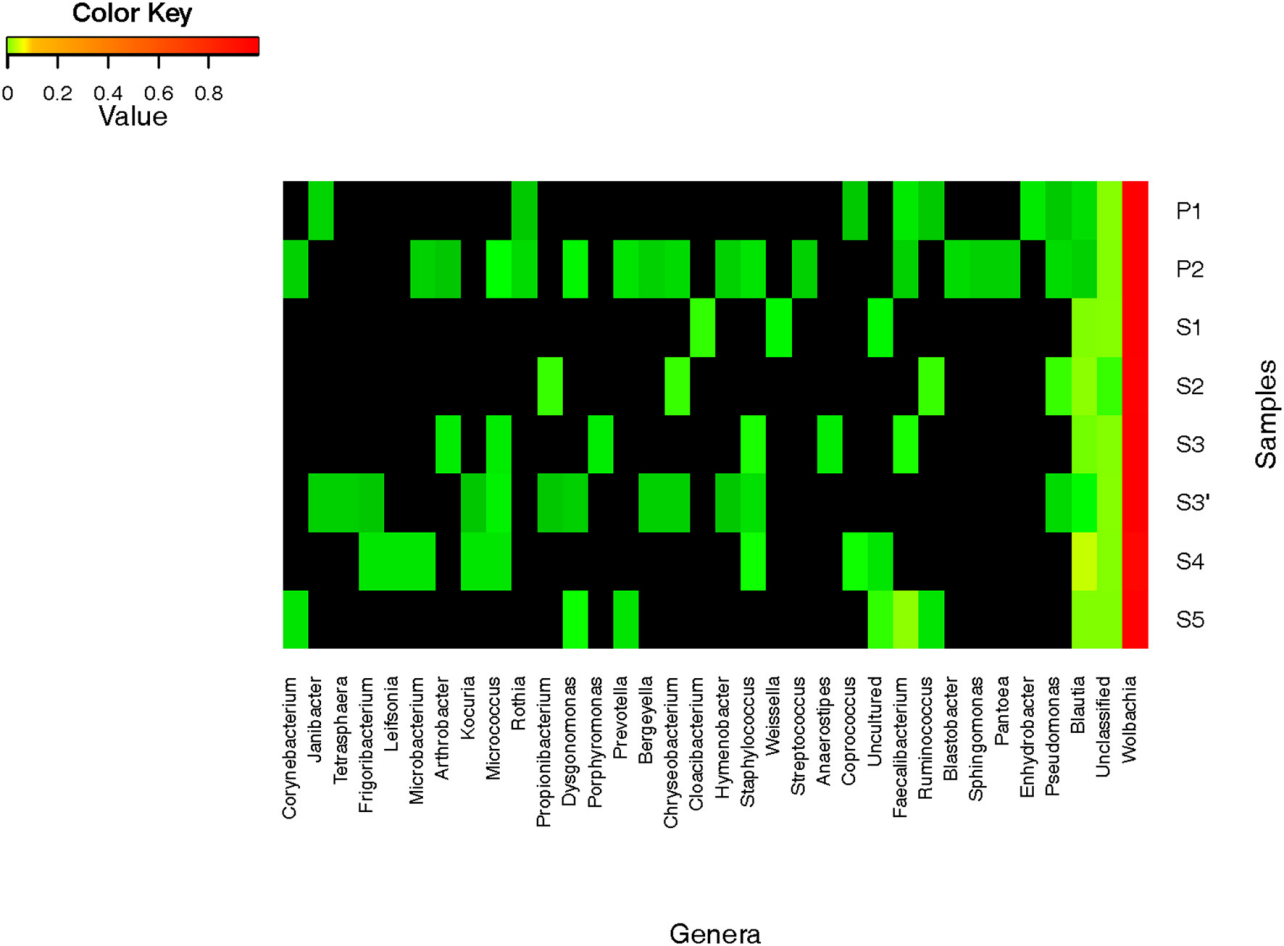
**Relative abundance of bacterial genera in *Aedes albopictus***. Heatmap represent the proportions of OTUs at the genus level.

A total of 417 OTUs were assigned to 4 phyla and 32 genera (Figure 4). Sequences from Proteobacteria are the predominant sequences found in all samples with all the experimental modalities tested (>99% of reads). About 96 to 99% of reads belonged to the *Wolbachia* genus and this largely explains the low diversity values obtained in each condition (Table 1). To confirm sequences assignment to *Wolbachia*, a local alignment of the two most abundant OTUs (Wolb G1 and Wolb G2) was performed against the non-redundant NCBI database to allow the selection of sequences from the most closely related taxa. The latter sequences were then aligned with Wolb G1 and Wolb G2 sequences and with 15 *Wolbachia* sequences retrieved from GenBank. This alignment highlighted the presence of a specific 25-bp sequence in the *Wolbachia rrs* gene (Figure S1). The alignment of this region with public databases was also performed using the SeqMatch tool (http://rdp.cme.msu.edu). The sequence showed 100% similarity and query coverage with other *Wolbachia* sequences but only 57% query coverage and 100% similarity with the nearest neighbor sequence from different taxa including *Candidatus mikurensis*, *Ehrlichia ruminantium*, and *Anaplasma platys*. This confirmed that the 25-bp sequence was specific to *Wolbachia*.

**Figure 5:**
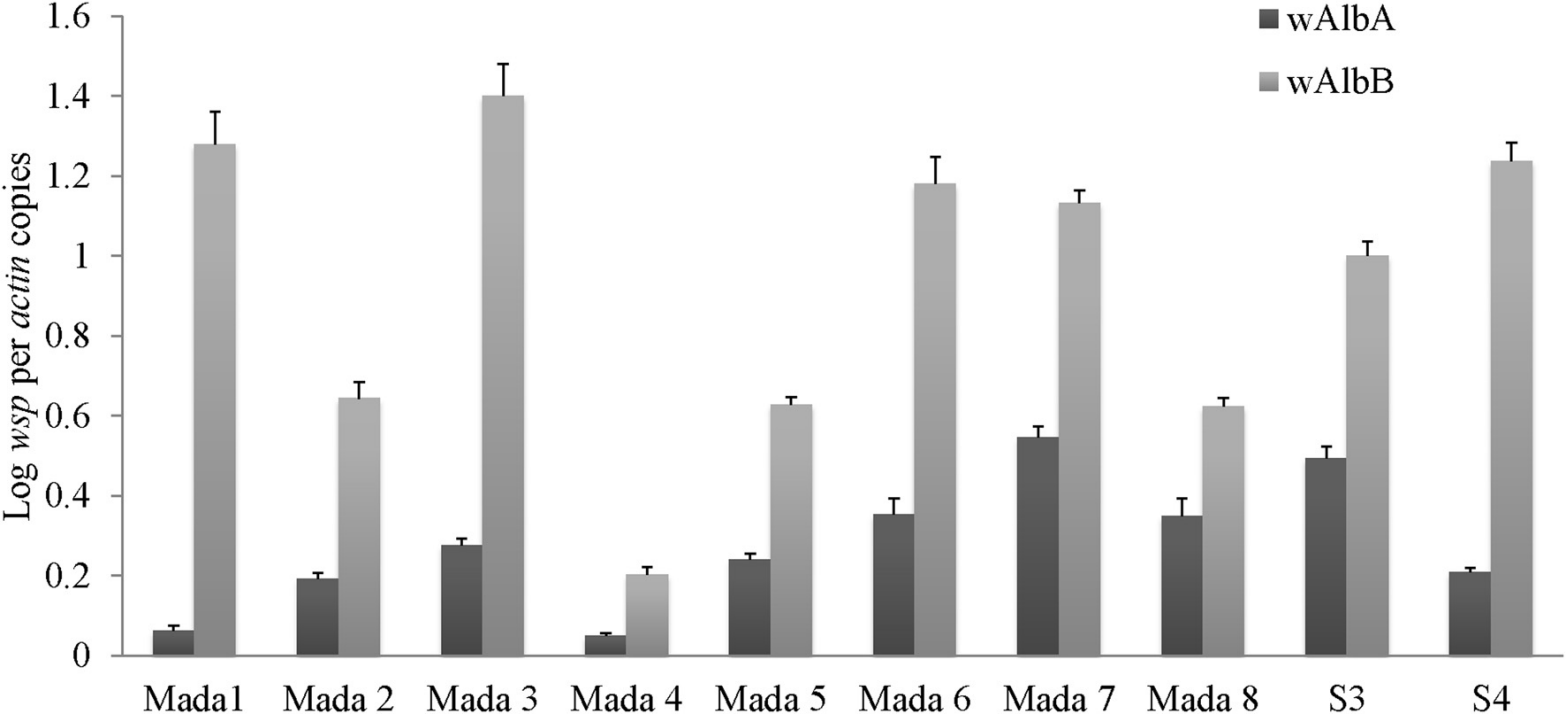
***Wolbachia* titers in *Aedes albopictus* individuals**. The ratio of *Wolbachia wsp* gene to *Aedes albopictus actin* gene copies is reported for *w*AlbA and *w*AlbB strains in S3 and S4 samples and for 8 other samples from the same site (Mada1 to Mada 8).

The numbers of *Wolbachia w*AlbA and *w*AlbB strains present in 10 mosquito individuals were quantified by clade-specific quantitative PCR of *wsp*, a gene encoding for *Wolbachia* surface protein, normalized to *actin*, a mosquito housekeeping gene. For each strain a relatively high gene copy number was found in all individuals tested (Figure 5). The *wsp*/*actin* ratios were between 6.25 × 10^0.01^ and 5.47 × 10^0.1^ for *w*AlbA and between 2.03 × 10^0.1^ and 1.4 × 10^1^ for *w*AlbB. Significant bacterial density differences were found between individual mosquitoes for both *w*AlbA (*p*-value = 8.499 × 10^−15^, Kruskall–Wallis test) and *w*AlbB (*p*-value = 3.616 × 10^−14^, Kruskall–Wallis).

### Taxonomic analysis and relative abundance of other bacterial taxa

Apart from *Wolbachia*, a total of 31 bacterial taxa were identified at the genus level from less than 1% of all the sequences (Figure 4). Firmicutes bacteria were found to be the dominant group (67%), followed by Actinobacteria (4%), Bacteroidetes (3%), and Proteobacteria (2%). In terms of OTU richness and genus content, ind and P2 were the most diverse with 22 and 19 genera identified, respectively (Tables 1, 2). Sequences corresponding to *Blautia*, a Firmicutes genus, were found in all the datasets from individual and pooled samples, ranging from 3.2% (for P1) to 89.2% (for S4) of sequences (Table 2). Moreover, 88 sequences from a single OTU were assigned to *Lachnospiaceae* family with no similarity at genus level. Overall, the proportion of sequences affiliated to known or unclassified bacterial genera varied among samples.

**Table 2:**
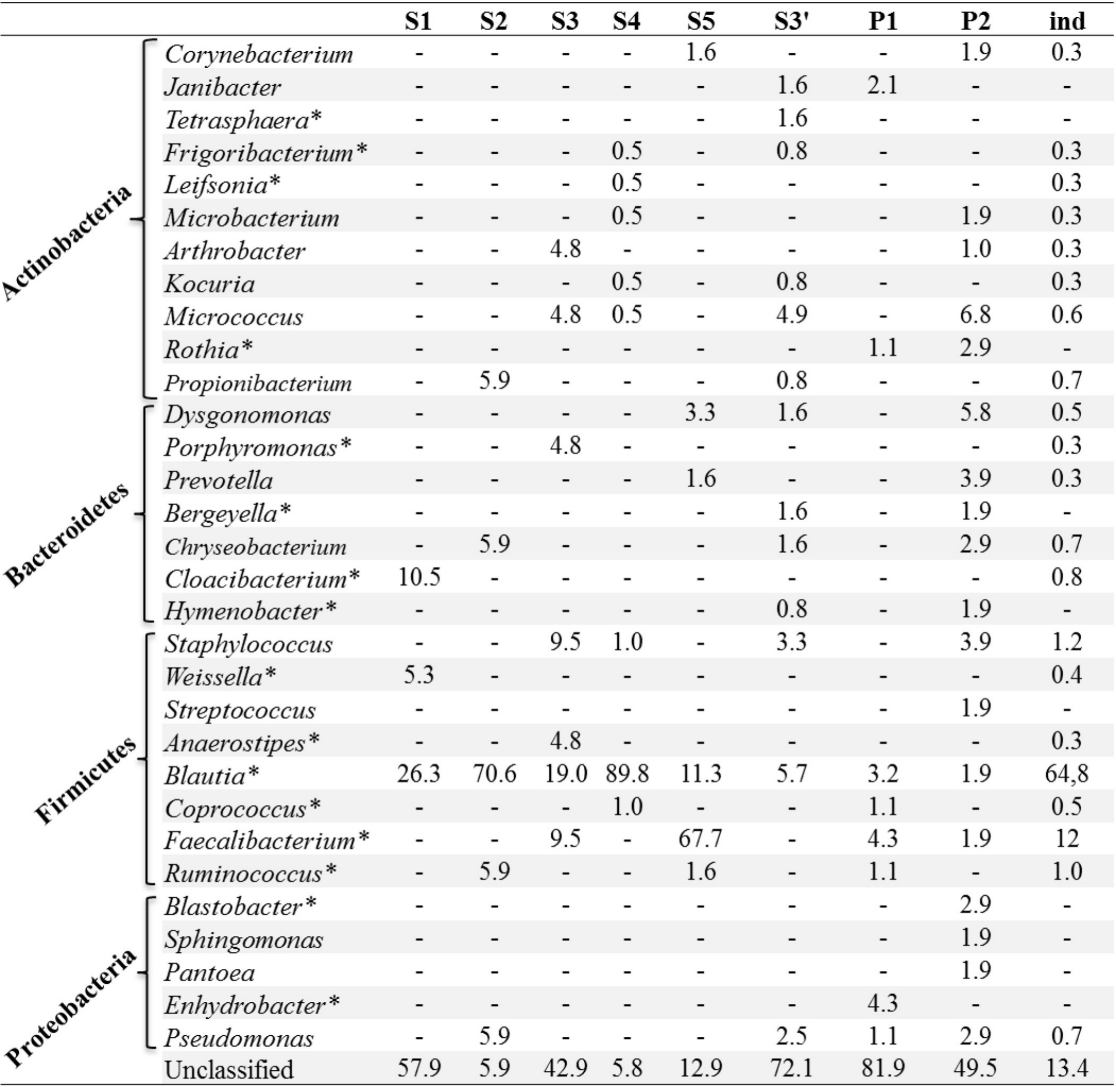
**Relative abundance of different genera without *Wolbachia***.

The asterisks (^*^) represent newly identified genera in mosquitoes according to Minard et al., 2013a,b.

## Discussion

The application of next-generation sequencing techniques to insect microbiota research makes it possible to gain a deeper knowledge of its microbial diversity. The goal of the present study was to assess the diversity of bacteria associated with the Asian tiger mosquito using high-throughput sequencing. Previous studies of bacterial diversity in various arthropods using a similar approach gave rise to different methodological parameters (Khöler et al., 2012; Moran et al., 2012; Osei-Poku et al., 2012; Ridley et al., 2012), so we chose to carry out a pilot study using pyrosequencing on a hypervariable region of 16S rRNA genes. Various experimental modalities were tested by varying the sample preparation (processing DNA samples from individuals or mixing several 16S amplicons either before or after PCR amplification) and depth of sequencing (number of reads). The primers chosen targeted the V5-V6 hypervariable region of 16S rRNA which allows a large number of bacterial taxa to be detected from sequences available in databases (94% of RDP database) and takes into account other limiting factors such as amplicon size and a lack of hybridization with host DNA.

The results obtained show first that a depth of at least 16,000 reads was necessary to accurately reach the saturation of richness discovery curves. Indeed, the observed richness was made to approach the estimated richness by increasing the depth of sequencing as demonstrated by comparing S3 (4276 reads) and S3′ (22,296 reads). In addition, as these two datasets originated from the same amplicon sample sequenced independently, the low Bray–Curtis dissimilarity levels between them reflects experimental reproducibility. Another factor evaluated was whether the pooling of samples would prove a cost-effective way to compare a huge number of samples. The drawback is that pooling may hide variability in the bacterial composition among samples. Here, we showed that it is preferable to pool amplicons rather than DNA before amplification as the latter method clearly failed to detect rare sequences.

In this study, pyrosequencing amplicons from *Ae. albopictus* bacterial microbiota revealed a lower Simpson diversity index than previously obtained with fingerprint methods (Zouache et al., 2011). However, using 454 pyrosequencing of 16S rRNA fragments, Osei-Poku et al. (2012) found that 23 genera occurred at a frequency of more than 1% in the guts of at least one individual of different mosquito species. They also reported very low bacterial diversity within individuals, with a single OTU typically making up two-thirds of all the bacteria in all field-caught individuals from seven species studied, which is similar to our results. Interestingly, the results presented here reveal the highest genus richness that we have yet found in *Ae. albopictus* using various methods (Zouache et al., 2011, 2012; Valiente Moro et al., 2013). The results show that high throughput sequencing is an efficient way to deeply investigate microbiota composition in mosquitoes.

We conclude that *Wolbachia* was the principle component of *Ae. albopictus* microbiota in different individuals as surprisingly about 99% of reads were *Wolbachia* sequences independent of the experimental method used. The relative densities of *Wolbachia w*AlbA and *w*AlbB in both field-caught and lab-reared *Ae. albopictus* have been measured by quantitative PCR and the values we report here are similar to those previously found in field-caught populations (Tortosa et al., 2010; Zouache et al., 2011, 2012). All mosquito samples analyzed here were positive for both strains. Possibly the abundance of *Wolbachia* and strain bi-infection confers a fitness advantage like that described in *Wolbachia*-superinfected mosquitoes (Dobson et al., 2002, 2004). This selective pressure that acts on hatching rates and lifespan in laboratory conditions could be a reason for the predominance of *Wolbachia* in field populations mitigating the metabolic cost of symbiosis. Cytoplasmic incompatibility induced by *Wolbachia* is also responsible for a high prevalence of superinfected females (Kittayapong et al., 2002; Dobson et al., 2004; Zouache et al., 2011).

Sequences affiliated to *Blautia* were the second most abundant after those from *Wolbachia*. This is the first report of the presence of this genus in *Ae. albopictus* and even in mosquitoes. *Blautia* are anaerobic acetogenic members of Firmicutes and some have been isolated from mammal guts (Bernalier et al., 1996; Gagen et al., 2010). In addition to *Blautia*, we discovered other anaerobic bacteria in *Ae. albopictus*, including *Faecalibacterium*, *Ruminococcus*, and *Coprococcus*. Some of these are thought to be involved in the metabolism of organic compounds such as acetic acid in the human gut (Tsai et al., 1976; Wrzosek et al., 2013). Of the 32 bacterial genera identified overall, 9 had been previously found in *Ae. albopictus* and 8 others had already been described in other mosquito species using low throughput molecular techniques (reviewed in Minard et al., 2013a). Among these bacteria, the genus *Pantoea* is one of the most prevalent bacterial cultivable groups identified in field-caught *Ae. albopictus* from Madagascar (Valiente Moro et al., 2013). The confirmation of the presence of these bacterial taxa in *Ae. albopictus* highlights the urgency of characterizing the metabolic aspects of these host-microbe interactions in relation to habitat specialization. Stinkingly, some human opportunistic pathogens belong to genera that were identified in mosquitoes such as *Pseudomonas, Staphylococcus, Chryseobacterium*, and *Dysgonomonas* (Hironaga et al., 2008; Asaad et al., 2013; Dettman et al., 2013; Ozcan et al., 2013; Coates et al., 2014). Linked to the contact between mosquito proboscis and human circulation during females' blood feeding, these observations argue the possibility of a mosquito-mediated bacterial transmission. Finally, a number of sequences were not assigned to any bacterial family but sequencing longer amplicons might improve their identification rate.

## Conclusion

Here we developed a high-throughput sequencing protocol to phylotype bacterial communities associated with *Ae. albopictus* mosquitoes. To our knowledge, this is the first time that such an approach has been used to explore *Ae. albopictus* microbiota. Although the results reveal the most complete picture of the bacterial microbiota in any given insect, they also show the limits of the high-throughput sequencing approach when there is a dominant endosymbiotic species. Alternative strategies such as the use of blocking primers (Vestheim et al., 2011) will be implemented to specifically discard sequences of the particularly abundant *Wolbachia* endosymbiont. The NGS approach developed in this study will be helpful to perform a large scale analysis of mosquito-associated microbiota from different geographic locations and would allow exploring the role of bacteria in invasive capacities of mosquitoes.

## Author contributions

Guillaume Minard, Claire Valiente Moro, and Patrick Mavingui conceived and designed the work. Guillaume Minard, Florence-Hélène Tran, and Van Tran-Van performed the experiments. Guillaume Minard and Audrey Dubost managed the data mining. Guillaume Minard, Claire Valiente Moro, and Patrick Mavingui analyzed and interpreted the data, and drafted the manuscript and the final version was approved by all the authors.

### Conflict of interest statement

The authors declare that the research was conducted in the absence of any commercial or financial relationships that could be construed as a potential conflict of interest.

## Supplementary material

Figure S1 **Alignment of *Wolbachia* reads**. *Wolbachia* reads Wolb G1 and Wolb G2 were aligned with sequences from most closely related taxa (based on V5-V6 *rrs* sequences) and other *Wolbachia* sequences retrieved from GenBank, using Jalview software. A 25-bp *Wolbachia* specific region is highlighted.
