# Supplementary material for: Pyrosequencing 16S rRNA genes of bacteria associated with wild tiger mosquito Aedes albopictus: a pilot study
Source: Front Cell Infect Microbiol. 2014 May 14;4:59. doi: 10.3389/fcimb.2014.00059 (PMC4030203; doi:10.3389/fcimb.2014.00059)
Supplement: Figure S1 — Alignment of Wolbachia reads. Wolbachia reads Wolb G1 and Wolb G2 were aligned with sequences from most closely related taxa (based on V5-V6 rrs sequences) and other Wolbachia sequences retrieved from GenBank, using Jalview software. A 25-bp Wolbachia specific region is highlighted. [file Presentation1.PPTX]

## Slide 1
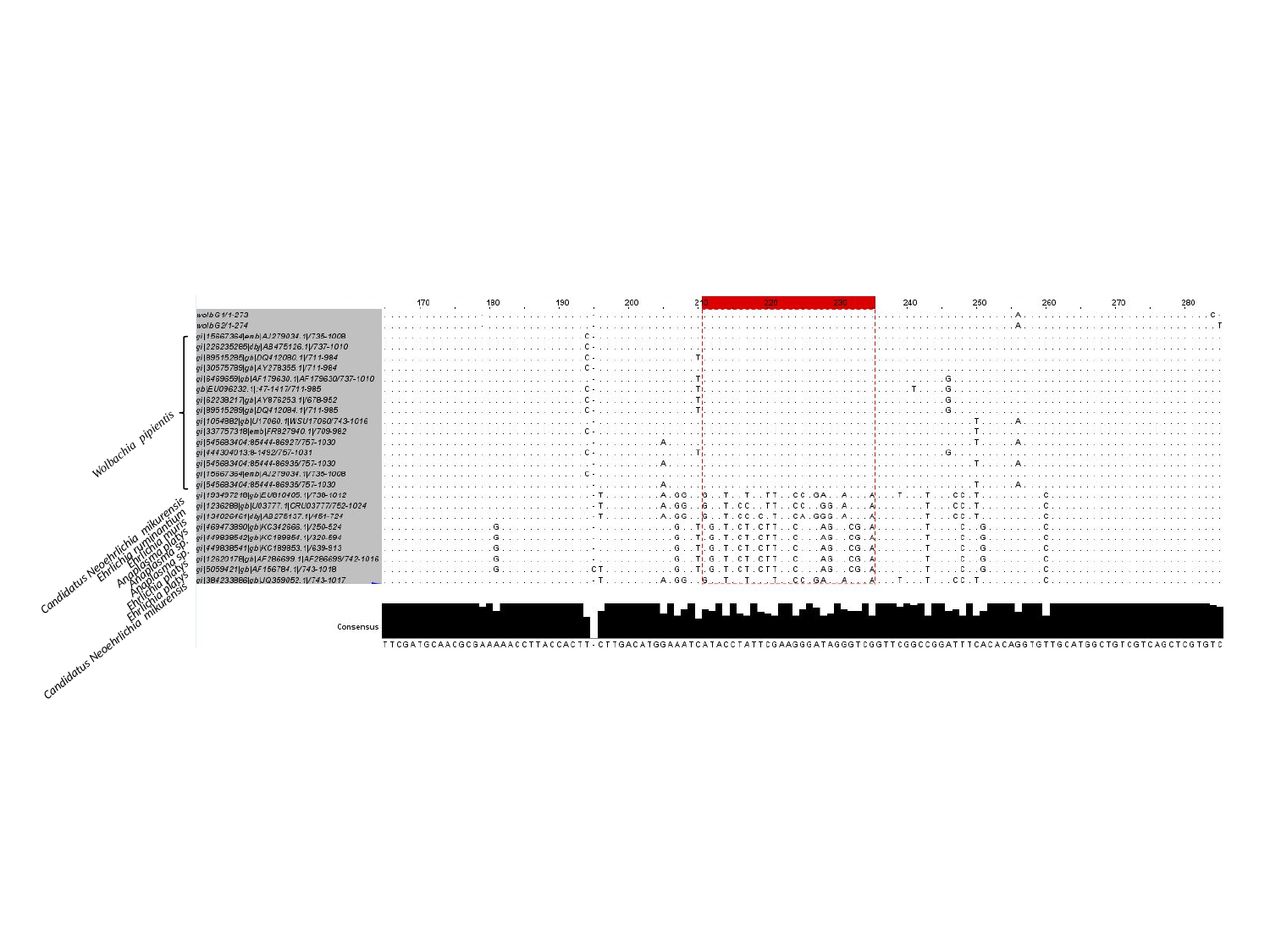

Wolbachia pipientis
Ehrlichia muris
Ehrlichia ruminantium
Anaplasma platys
Anaplasma sp.
Anaplasma sp.
Ehrlichia platys
Candidatus Neoehrlichia mikurensis
Ehrlichia platys
Candidatus Neoehrlichia mikurensis
